# Using empirical biological knowledge to infer regulatory networks from multi-*omics* data

**DOI:** 10.1186/s12859-022-04891-9

**Published:** 2022-08-22

**Authors:** Anna Pačínková, Vlad Popovici

**Affiliations:** 1 RECETOX, Faculty of Science, Masaryk University, Kotlarska 2, Brno, Czech Republic; 2 Faculty of Informatics, Masaryk University, Botanicka 68a, Brno, Czech Republic

**Keywords:** Integrative analysis, Multimodal omics, Bayesian networks, Regulatory networks, Knowledge discovery

## Abstract

**Background:**

Integration of multi-*omics* data can provide a more complex view of the biological system consisting of different interconnected molecular components, the crucial aspect for developing novel personalised therapeutic strategies for complex diseases. Various tools have been developed to integrate multi-*omics* data. However, an efficient multi-*omics* framework for regulatory network inference at the genome level that incorporates prior knowledge is still to emerge.

**Results:**

We present IntOMICS, an efficient integrative framework based on Bayesian networks. IntOMICS systematically analyses gene expression, DNA methylation, copy number variation and biological prior knowledge to infer regulatory networks. IntOMICS complements the missing biological prior knowledge by so-called *empirical* biological knowledge, estimated from the available experimental data. Regulatory networks derived from IntOMICS provide deeper insights into the complex flow of genetic information on top of the increasing accuracy trend compared to a published algorithm designed exclusively for gene expression data. The ability to capture relevant crosstalks between multi-*omics* modalities is verified using known associations in microsatellite stable/instable colon cancer samples. Additionally, IntOMICS performance is compared with two algorithms for multi-*omics* regulatory network inference that can also incorporate prior knowledge in the inference framework. IntOMICS is also applied to detect potential predictive biomarkers in microsatellite stable stage III colon cancer samples.

**Conclusions:**

We provide IntOMICS, a framework for multi-*omics* data integration using a novel approach to biological knowledge discovery. IntOMICS is a powerful resource for exploratory systems biology and can provide valuable insights into the complex mechanisms of biological processes that have a vital role in personalised medicine.

**Supplementary Information:**

The online version contains supplementary material available at 10.1186/s12859-022-04891-9.

## Introduction

The rapid development of high-throughput technologies has led to large production and availability of *omics* data. Single-*omics* technologies measure simultaneously molecules of the same type from biological samples. On the contrary, multi-*omics* data collect multiple modalities from the same set of samples and describe different aspects of cellular functioning. Therefore, multi-*omics* data contain complementary information and provide a holistic view of the biological system consisting of different interconnected molecular components.

Integration of multi-*omics* data can enhance our understanding of biological systems, crucial for developing novel personalised therapeutic strategies for complex diseases. Hence, developing a computational framework to infer regulatory relationships by integrating multiple modalities is one of the most relevant and challenging problems in systems biology.

The regulatory network inference from high-throughput data is limited by noise or measurement errors. However, it could be significantly improved by incorporating a wealth of biological prior knowledge from the scientific literature [1, 2]. Gene regulatory networks inferred from single-*omics* data (gene expression) and prior knowledge were frequently used to model gene-gene interactions [1, 3–5]. However, the flow of genetic information in biological systems is very complex, and gene expression is a product of multiple biological processes and control mechanisms, such as copy number variations (CNVs), transcription factors (TFs), non-coding RNAs, DNA methylations, or histone modifications. TFs bind to regulatory elements in the promoter of a given gene and initiate and regulate its transcription [6]. Copy number variations such as amplifications/deletions of a DNA segment can affect gene expression through simple gene dosage effects and result in the overexpression/silencing of given genes [7]. DNA methylation of the promoter region is known to down-regulate gene expression by preventing the binding of transcription factors [8]. However, plenty of studies suggest that DNA methylation and its effect on gene expression needs to be interpreted differently in particular regions of the gene body [9–11]. Regarding all these aspects, it is crucial to progress from single-*omics* data analysis and derive causal relationships between features from multi-*omics* data.

There are several tools estimating the dependence structure among multi-*omics* data [12, 13], estimating a large number of networks for each gene where different modalities are treated as nodes in a graphical model. Although this approach provides valuable insights, they are limited and should be complemented with gene-gene interactions. Several other tools for gene regulatory network inference from multi-*omics* data based on correlation or regression were proposed [14–16]. However, these tools do not integrate biological prior knowledge from the databases, and their main limitation is missing implementation.

[17] propose a framework to identify disease-specific pathways by integrating gene expression, mutation information and prior knowledge through a Bayesian network. One of its drawbacks is data discretisation, which implies substantial information loss. RACER [18] models the gene expression as response using transcription factor (TF) data, CNV, DNA methylation, and micro RNA (miRNA) expression signals as explanatory variables. RACER applies a two-stage regression framework: first infers the sample-specific regulatory activities by TFs and miRNAs, which are then used as inputs to infer specific TF/miRNA-gene interactions.

During the last year, some up-and-coming tools were published for integrative analysis of multi-*omics* data utilising prior knowledge to infer regulatory networks. COSMOS [19] developed a systematic approach to search public databases for plausible causal links between significantly deregulated TFs, kinases/phosphatases and metabolites. The prior knowledge from differential analysis is then used to systematically search causal paths between the deregulated TFs, kinases/phosphatases, and metabolites using a CARNIVAL and its integer linear programming optimisation approach [20]. To derive mechanistic hypotheses for experimental observations using COSMOS, we need a case-control study to perform differential analysis, and these data may not always be available. KiMONo [21] optimises sparse group LASSO (SGL; LASSO least absolute shrinkage and selection operator) penalisation [22] in the multivariate regression to model gene expression of each gene separately. The gene expression represents the criterion variable, and the input matrix is assembled by the features associated with the gene within the prior. SGL penalises within and between predefined groups of features (the authors call it ‘bi-level’ selection), enabling KiMONo to account for different underlying distributions between the features originating from multi-*omics* data. Finally, these fitted models are aggregated in the final heterogeneous multi-*omics* network. CANTARE [23] focuses mainly on relationships between omics modalities. CANTARE fits pairwise regression models across all pairs of *omics* data resulting in the network. The relationships from the resulting network are then utilised with other variables to predict the outcome by predictive logistic regression models.

Motivated mainly by [3, 4], we present IntOMICS, a novel Bayesian framework for multi-*omics* data integration using prior knowledge to infer regulatory networks. Even if the intensive research has deposited a wealth of biological prior knowledge into public databases, some regulatory events between genes are still missing. Although databases such as DbVar [24] or iMETHYL [25] exist, a database with known CNV/METH and gene expression interactions is missing. Therefore, IntOMICS incorporates a novel approach to biological knowledge discovery—estimates the *empirical* biological knowledge to complement the available data from public databases. IntOMICS is designed to capture relevant crosstalks within and between copy number variation, DNA methylation and gene expression. The model parameters tuning guarantees accurate model design and robust results inference.

The performance of proposed algorithm at the multi-*omics* level is compared with RACER and KiMONo, algorithms that can also incorporate prior knowledge in the inference framework.

Werhli and Husmeier [4] algorithm (W &H) represents one of the most relevant gene regulatory network reconstruction tools based on Bayesian networks. Therefore, the W &H algorithm is selected for performance comparison with IntOMICS at the gene expression level. Both algorithms resemble the core formulation of prior distribution and integration of biological prior knowledge. On the contrary, they differ in two key aspects: (i) IntOMICS combines prior knowledge with data-derived evidence—the *empirical* biological knowledge (ii) IntOMICS is designed to infer not only dependencies among gene expression but also between gene expression, DNA methylation and copy number variation. W &H algorithm relies on conventional MCMC sampling, which tends to be slow in convergence and mixing and can often be stuck in low-probability regions. The inclusion of adaptive MCMC simulation and Markov blanket resampling (MBR) [26] minimise the weaknesses of the W &H algorithm.

IntOMICS can theoretically be extended with any additional modality if the proposed model assumptions are satisfied. We assume that variables come from the multivariate Gaussian distribution, so no discretisation is needed. In addition, only biologically relevant dependencies respecting the central dogma of molecular biology must be considered.

## Bayesian networks

A Bayesian network (BN) is a graphical model representing probabilistic relationships among random variables. BN is defined by the joint probability distribution over the variables specified by (i) a network structure G represented by a directed acyclic graph (DAG) with a set of nodes (indicating a set of random variables) and a set of directed edges (indicating conditional dependence relations among random variables), and (ii) a family of conditional probability distributions with corresponding parameters characterising the dependencies represented by the set of edges.

Due to the Markov condition (each variable is conditionally independent of the set of all its non-descendants given the set of all its parents), the joint probability distribution factorises as follows:

1 $$\begin{aligned} P(X_1,\ldots ,X_N | G) = \prod \limits _{j=1}^N P(X_j|X_{pa_j(G)}), \end{aligned}$$

where $X_1,\ldots ,X_N$ are random variables, $X_{pa_j(G)}$ are parents of $X_j$ implied by the network structure G.

Learning the Bayesian network structure from the data is one of the most challenging tasks. It is guided by a scoring metric *S*, which assess the agreement between a given network structure and the available data *D*. The aim is to identify the highest-scoring network structure.

$S$ is proportional to the posterior probability of the network structure given the data $D$ and factorises into a product where each term depends only on a given node and its parents:

2 $$\begin{aligned} P(G | D) \propto P(D | G) P(G) = \prod \limits _{j=1}^N S(X_j, X_{pa_j} | D ), \end{aligned}$$

The choice of scoring metric either requires the data to be discretised (BDe score, [27]) or can only capture linear regulatory relationships (BGe score, [28]). The BGe score is developed for continuous data sampled from a multivariate normal distribution. Since gene expression, copy number variation, and DNA methylation data are continuous, and we want to avoid data discretisation because of information loss, we consider BGe scoring metric. Furthermore, it has been shown that BGe remains as powerful as BDe, even in the case of slight departures from the linearity assumption [29].

## Methods

**Fig. 1:**
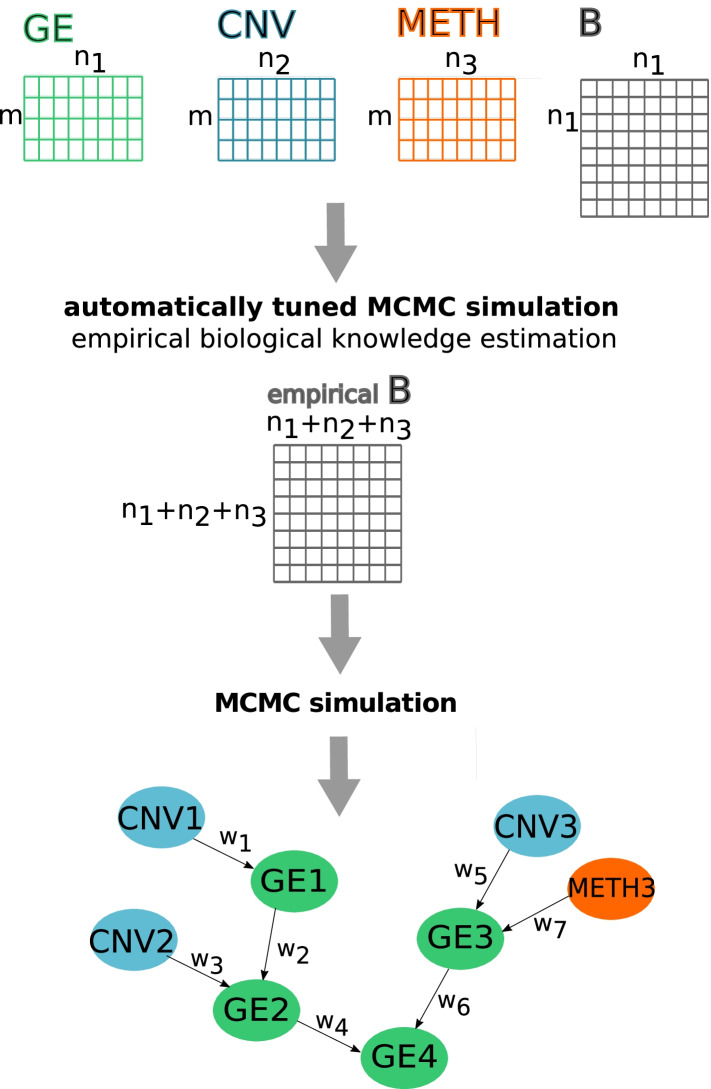
IntOMICS framework. IntOMICS framework takes as input (i) gene expression matrix *GE* with $m$ samples and $n_1$ genes, (ii) the associated copy number variation matrix $CNV$ ($m$ x $n_2$), (iii) the associated DNA methylation matrix of beta-values $METH$ ($m$ x $n_3$) sampled from the same individuals, and (iv) the biological prior knowledge matrix $B$ ($n_1$ x $n_1$) with information on known interactions among molecular features. An automatically tuned MCMC algorithm [30] estimates parameters and *empirical* biological knowledge. Conventional MCMC algorithm with additional Markov blanket resampling step is used to infer resulting regulatory network structure consisting of three types of nodes: GE nodes (highlighted in green) refer to gene expression levels, CNV nodes (highlighted in blue) refer to copy number variations, and METH nodes (highlighted in red) refer to DNA methylation. Edge weight $wi$ represents the empirical frequency of given edge over samples of network structures

We present IntOMICS, a powerful Bayesian framework for multi-*omics* data integration to capture dependencies among different molecular features. Figure 1 summarises the key steps of our novel framework.

IntOMICS integrates the biological knowledge from databases and is designed to capture relevant crosstalks within and between gene expression, copy number variation and DNA methylation. The missing biological prior knowledge is complemented by so-called *empirical* biological knowledge, estimated from the available experimental data. The *empirical* biological knowledge reflects hidden patterns in the available datasets derived from past iterations of the Markov chain.

The proposed framework avoids data discretisation, which implies substantial information loss. IntOMICS parameters tuning guarantees accurate model design and robust results inference. The inclusion of adaptive MCMC simulation and Markov blanket resampling (MBR) [26] improves convergence and mixing of the Markov chain.

### Biological prior knowledge integration

Biological prior knowledge with information on known interactions among molecular features is integrated into the regulatory network inference employing a prior probability of the network structure G. As in [3, 4], we define the prior distribution over network structures as:

3 $$\begin{aligned} P(G|\beta ) = \frac{e^{-\beta E(G)}}{{Z(\beta )}} = \frac{e^{-\beta E(G)}}{\sum \limits _{G \in {\mathcal {G}}} e^{-\beta E(G)}}, \end{aligned}$$

where ${\mathcal {G}}$ indicates a set of all possible network structures, and the parameter $\beta$ controls the strength of the influence of the biological prior knowledge. The energy function E(G) measures the agreement between the biological prior knowledge and the current network structure:

4 $$\begin{aligned} E(G) = \sum \limits _{j=1}^N \varepsilon (X_j, X_{pa_j(G)}), \end{aligned}$$

5 $$\begin{aligned} \varepsilon (X_j, X_{pa_j(G)}) = \sum \limits _{i \in X_{pa_j}} (1-B_{ij}) + \sum \limits _{i \notin X_{pa_j}} B_{ij}, \end{aligned}$$

where *B* is adjacency matrix with $B_{ij} \in [0,1]$. The matrix *B* represents the biological prior knowledge with information on known interactions among molecular features. If there is the prior knowledge about the direct interaction from node *i* to node *j*, $B_{ij} = 1$. If there is the prior knowledge about the interaction of a transcription factor *i* and its target *j*, $B_{ij} = 0.75$. If there is no knowledge about the direct interaction from node *i* to node *j*, $B_{ij} = 0.5$. If we have prior knowledge that there is no edge from node *i* to node *j*, $B_{ij} = 0$.

Then, from (4) we obtain the upper bound of the partition function $Z(\beta )$ (see [4]):

6 $$\begin{aligned} Z(\beta ) = \prod \limits _{j} \sum \limits _{X_{pa_j}} e^{-\beta \varepsilon (X_j, X_{pa_j(G)}) }. \end{aligned}$$

In the current experiment, curated regulatory relationships of publicly available network database KEGG [31] and target gene-transcription factor associations database ENCODE [32–34] are used as prior knowledge. Nevertheless, our approach is not limited to KEGG/ENCODE, any other available database can be used.

### MCMC sampling scheme for Bayesian network structure learning

A complete comparison of posterior probabilities is intractable since the search space of all possible network structures grows super-exponentially with the number of random variables. Hence, a Markov Chain Monte Carlo (MCMC) sampling scheme [35] is adopted to generate a sample of network structures from the posterior distribution.

First, we sample a network structure $G_c$ while keeping the $\beta$ parameter fixed. We need to define the proposal distribution $Q(G_c|G_s)$ to draw candidate network structures from an intractable posterior distribution. The candidate network structure $G_c$ is proposed by either adding, deleting or reversing a particular directed edge from the current network structure $G_s$. Besides a conventional single edge proposal move, the Markov blanket resampling step [26] is introduced with a fixed probability $p_{MBR}~=~1/15$ to achieve faster mixing and convergence ($p_{MBR}$ suggested by the authors). The acceptance probability *A* indicates how probable the candidate network structure is with respect to the current network structure, according to the posterior distribution. A candidate network structure proposed from the proposal distribution $Q(G_c|G_s)$ is accepted according to the Metropolis-Hastings rule [36] with the acceptance probability given by:

7 $$\begin{aligned} A = \min \left\{ \frac{P(D|G_c)P(G_c|\beta )Q(G_s|G_c)}{P(D|G_s)P(G_s|\beta )Q(G_c|G_s)},1\right\} . \end{aligned}$$

After each iteration except the sampling phase (see “Technical details” section), a new parameter $\beta _c$ for fixed network structure *G* is proposed and accepted according to the following acceptance probability:

8 $$\begin{aligned} A_\beta = \min \left\{ \frac{P(G|\beta _c)}{P(G|\beta _s)},1\right\} , \end{aligned}$$

where $\beta _s$ refers to the current parameter value.

### Technical details

The main steps of IntOMICS are summarised in Algorithm 1.

IntOMICS framework takes as input (i) gene expression matrix *GE* ($m$ x $n_1$), (ii) the associated copy number variation matrix $CNV$ ($m$ x $n_2$), (iii) the associated DNA methylation matrix of beta-values $METH$ ($m$ x $n_3$) sampled from the same individuals, and (iv) the biological prior knowledge matrix $B$ ($n_1$ x $n_1$) with information on known interactions among molecular features.

DNA methylation is an epigenetic mechanism involving the transfer of a methyl group in CG dinucleotides (CpGs). DNA methylation microarrays use beads with long target-specific probes designed to capture individual CpG sites. Because multiple CpG sites are associated with a single gene, we can use linear regression to detect relevant probes that are associated with the gene expression. If not stated otherwise, we considered individual probes with a statistically significant coefficient (*p*-value $< 0.5$) and $R^2 > 0.3$ as possible regulators of given gene expression. The ordered quantile normalisation [37] is used to transform DNA methylation beta-values to Gaussian distribution.

Adaptive MCMC algorithms use information from past iterations of the chain to improve computational efficiency. We use an automatically tuned MCMC algorithm [30] with default hyper-parameters to estimate parameter $\beta$ and *empirical* biological knowledge matrix ${\mathcal {B}}$ through multiple phases. The automatically tuned MCMC algorithm consists of several distinct phases.

The first adaptive phase is used to roughly tune the parameter $\beta$, more precisely the variance of its proposal distribution $\sigma _s^2$. The proposal distribution is $\beta _c~\sim ~N(\beta _s,\sigma _s^2)$, where $\beta _s$ refers to the current parameter value, and $\beta _c$ refers to the candidate parameter value. The initial value of the parameter $\beta _s$ is randomly drawn from *U*[0, 10], and then we require $\beta >= 0.5$. The initial value $\sigma _s = 5$.

The transient phase is applied to diagnose whether the chain has reached the mode of the target distribution.

The second adaptive phase is used to fine-tune the variance $\sigma _s^2$, $\beta$ parameter estimation and compute the *empirical* biological prior matrix ${\mathcal {B}}$. Assuming $B_{ij} = 0.5$, the prior knowledge about interaction from node *i* to node *j* is updated during the second adaptive phase after every conventional single edge proposal move. The ${\mathcal {B}}_{ij}$ value corresponds to the ratio of acceptance (number of iterations with accepted candidate edge from node *i* to node *j*) and frequency (number of iterations with proposed candidate edge from node *i* to node *j*) (for details, see Table 1). Reversing an edge is equivalent to deleting the edge and adding the edge in the opposite direction.

**Table 1:** Empirical biological knowledge estimation

Edge	Operation	Frequency	Candidate	Acceptance
$G_i$ $G_j$	Add	$f_{ij} = f_{ij} + 1$	Accepted	$a_{ij} = a_{ij} + 1$
			Rejected	$a_{ij} = a_{ij}$
$G_i$ $G_j$	Delete	$f_{ij} = f_{ij} + 1$	Accepted	$a_{ij} = a_{ij}$
			Rejected	$a_{ij} = a_{ij} + 1$
$G_i$ $G_j$	Reverse	$f_{ij} = f_{ij} + 1$	Accepted	$a_{ij} = a_{ij}$
				$$a_{ji} = a_{ji} + 1$$
		$f_{ji} = f_{ji} + 1$	Rejected	$a_{ij} = a_{ij} + 1$
				$$a_{ji} = a_{ji}$$

Assuming there is no prior knowledge about the direct interaction from node *i* to node *j*, the *empirical* biological matrix ${\mathcal {B}}$ is estimated, with ${\mathcal {B}}_{ij} = \frac{a_{ij}}{f_{ij}} \in [0,1]$

The *empirical* biological matrix ${\mathcal {B}}$ and the parameter $\beta$ determined by the second adaptive phase are used in the last sampling phase. In this phase, IntOMICS applies a *greedy horizon* approach. Three independent paths are executed with a fixed BGe score (except the MBR step). The most probable path is chosen after every 500 iterations. In our simulation, two independent samples of network structures are produced. Each sample consists of 200,000 DAGs (with a burn-in period of 100,000 iterations). The resulting samples of DAGs are thinned—discarded all but every 500th DAG. The burn-in period of 100,000 iterations and thinning frequency of 500 are arbitrary choices. We tested different settings of these parameters using in silico dataset with known network structure but they did not influence the resulting accuracy (see Additional file 1: Figs. S1 and S2).

Distinct DAGs can describe the same set of independence relations and have the same likelihood score. Such DAGs are from the same equivalence class. The equivalence class can be represented by completed partially directed acyclic graphs (CPDAGs). Therefore, we convert DAGs into corresponding CPDAGs and discard duplicated CPDAGs.

The convergence of resulting Markov chains is examined using the $c_{\mathop {rms}}$ measure [38]. The $c_{\mathop {rms}}$ threshold is given by the third quartile of $|c_{\mathop {rms_k}} - c_{\mathop {rms_{k-1}}}|$ for each iteration *k*. If the $c_{\mathop {rms}}$ value of the last 100 iterations (after thinning) is smaller than the $c_{\mathop {rms}}$ threshold, the MCMC simulation stops. Otherwise, the simulation proceeds until this condition is met. Subsequently, we discard the CPDAGs from the burn-in period.

The strength of the probabilistic relationships expressed by the edges in the resulting network structure is measured as their empirical frequency over both independent sets of CPDAGs.

In the context of gene expression, gene transcription is usually controlled by a small number of transcription factors. In contrast, a transcription factor can control an almost unlimited number of genes. Therefore, we apply the upper bound of the number of parents for each GE node: each GE node can have at most three GE parent nodes and one corresponding CNV node. There is no restriction on the number of METH parents for given GE node. CNV and METH nodes cannot have any parents. This restriction is supported by the biological literature [39].

Analyses were carried out using the free R software (www.r-project.org) version 4.0.0.
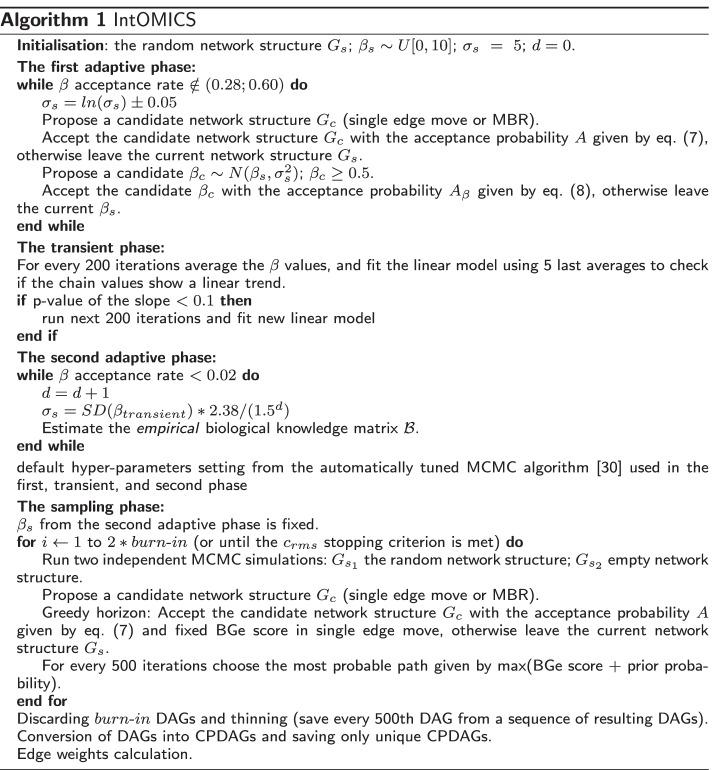


## Data and test procedures

Objective assessment of regulatory network inference is challenging [40], especially if we are interested in multiple molecular features, such as gene expression, copy number variation, and DNA methylation.

DREAM (the Dialogue on Reverse Engineering Assessment and Methods) project [40] was established to objective assess network inference methods on benchmark data sets. We use one of the DREAM4 gene expression datasets [41–43] for the IntOMICS evaluation at gene expression level. But we must keep in mind, that simulated datasets cannot fully reflect underlying biological processes.

We use also several real datasets to evaluate the IntOMICS performance considering multi-*omics* data. There are multiple publicly available databases with well-known interaction pathways. We can use them as the ground truth. Nevertheless, a certain level of disagreement is likely to emerge due to biological diversity. Well-known interaction pathways capture only simplified representations of mechanisms ongoing in most populations under given conditions. As a test case for IntOMICS, we focus mainly on the Wnt/Wingless and MAPK signalling pathways that have a prominent role in cancer development. Hence, they have been extensively studied in the context of colon cancer, and most of the key gene-gene interactions have been revealed and experimentally tested [44–47]. Besides wild-type/normal tissue experiments, we assess the IntOMICS performance in samples affected by some perturbation (interventional gene expression data from knock-out experiments, tumour tissues).

DNA methylation is one of the most intensely studied epigenetic modifications of DNA that is important for the normal regulation of transcription, embryonic development, genomic imprinting, genome stability and chromatin structure [48]. The Beta-value (frequently converted to M-value through a logistic transformation) is a metric to measure DNA methylation levels. The Beta-value ranges between 0 (completely unmethylated) and 1 (every copy of the site was methylated) and follows the Beta distribution [49]. The copy number variations contain information on gains and losses of genetic information. CNV data are represented by continuous segment mean values reflecting the log2 ratio of probe intensities. Diploid regions have a segment mean of zero, amplified regions have positive values, and deletions have negative values [50].

### Datasets

A summary of all data sets used in this study can be found in Table 2.

**Table 2:** Summary of all used data sets

Dataset	Investigated gene set	Samples	Details
GSE127960 ZIC5 WT	16GE / 0CNV / 0METH	2	HCT116 colon cancer cell line
GSE127960 ZIC5 KO	16GE / 0CNV / 0METH	4	HCT116 colon cancer cell line
			ZIC5 knockout
TCGA-COAD MSS	24GE / 24CNV / 4METH	27	Primary tumour stage II/III
			With MSS phenotype
TCGA-COAD MSI	24GE / 24CNV / 3METH	69	Primary tumour
			With MSI phenotype
TCGA-COAD NAT	24GE / 24CNV / 16METH	19	Histologically normal tissue
			Adjacent to the tumour
DREAM4 1-5	10GE / 0CNV / 0METH	1	Five independent in silico networks
			To assess the consistency of prediction
TCGA-AML	25GE / 25CNV / 25METH	173	Acute myeloid leukemia
PETACC-3 MSS	39GE / 23CNV / 0METH	176	Primary tumour stage III
			With MSS phenotype

*MSS* microsatellite stability, *MSI* microsatellite instability, *NAT* histologically normal tissue adjacent to the tumour, *GE* gene expression, *CNV* copy number variation, *METH* methylation probe, *KO* knockout, *WT* wild-type, *AML* acute myeloid leukemia

The Cancer Genome Atlas (TCGA) [51] provides publicly available multi-*omics* datasets for human cancers, including colon cancer (COAD) and its histologically normal tissue adjacent to the tumour (NAT). The copy number variation of the associated genes from TCGA-COAD samples were downloaded from the Broad Institute GDAC Firehose (https://gdac.broadinstitute.org/; accessed 30 December 2020). We use only samples with DNA methylation data from Illumina Infinium HumanMethylation450 (450K) BeadChip array available. The resulting subset of the TCGA-COAD consists of n = 115 samples (27 microsatellite stable phenotype (MSS) stage II/III, 69 microsatellite instable phenotype (MSI), 19 NAT). Several TCGA-COAD samples lack information on microsatellite status. These samples were classified into MSI/MSS groups using MSI gene expression signature [52].

GSE127960 includes the gene expression profiling of the human colon cancer cell lines HCT116 with ZIC5 wild-type (ZIC5 WT) and ZIC5 knockout (ZIC5 KO) replicates.

DREAM4 dataset [41–43] consists of five in silico networks with 10 nodes. We use steady state data reflecting gene expression measurements.

We utilise another dataset originating from TCGA [51]. We downloaded the processed TCGA-AML (acute myeloid leukemia) dataset used in the original RACER publication [18] to reproduce the most relevant results. TCGA-AML dataset consists of gene expression, copy number variation, DNA methylation and miRNA expression data. Methylation data are represented by the mean of multiple probes corresponding to a given gene.

PETACC-3 clinical trial [53, 54] investigates the benefit of irinotecan in the adjuvant treatment of stage III colon cancer patients. We use gene expression and copy number variation data of 176 MSS stage III colon cancer samples (PETACC-3 MSS). Missing MSS/MSI phenotype was determined by the MSI gene expression signature mentioned above.

### Evaluation criteria

One of the main performance indexes is used the receiver-operating characteristic curve (ROC) as a function of the edge weights and area under the receiver-operating characteristic curve (AUC). Edge weight in the resulting network structure reflects its empirical frequency over the final set of CPDAGs.

From a practical point of view, the edge weight expresses confidence in the real edge presence. If not stated otherwise, we define a threshold for a presence of an edge as 0.75 quantile of all present edges in a given network structure.

The IntOMICS performance at gene expression level is compared with the W &H algorithm—one of the most relevant gene regulatory network reconstruction tools based on Bayesian networks [4]. The W &H algorithm was designed to infer only dependencies among gene expression data. Thus, we exclude CNV-GE and METH-GE edges identified by IntOMICS in the performance comparison of these two algorithms. The criterion of the 83% confidence intervals (CIs) overlap [55] is used to test if there is a difference in the performance of both algorithms. To measure the agreement between IntOMICS and W &H algorithm, we add another performance metric—Cohen’s $\kappa$ coefficient. To assess which of the tested algorithms estimate more biologically relevant gene-gene interactions, we define a set of edges (missing in the prior knowledge) identified exclusively by IntOMICS or W &H, respectively, and compare them against other publicly available interaction databases [56–58].

To evaluate the IntOMICS performance in real datasets, we select the Wnt signalling pathway from the KEGG database and consider only interactions experimentally validated by low-throughput experiments listed in the BioGrid database [57]. We refer to this pathway as the *gold standard* Wnt pathway (GS Wnt pathway). GS Wnt pathway includes only interactions with strong experimental support but includes many false negatives. The missing interactions in the GS Wnt pathway are not necessarily incorrect. Regulatory networks derived using TCGA-COAD MSI/NAT, and GSE127960 ZIC5 WT/KO are compared with the GS Wnt pathway used as the ground truth.

$\beta$-*catenin regulation in MSI colon cancer* Activating mutations in $\beta$-catenin (CTNNB1) result in decreased phosphorylation by GSK3$\beta$ and increased Wnt signalling through the Tcf/Lef transcription factors. These mutations are more frequent in microsatellite instable (MSI) colon cancer [59–61]. KEGG Colorectal cancer pathway has MSI specific information on missing interaction between GSK3$\beta$ and CTNNB1 genes. Therefore, we compare regulatory networks inferred by IntOMICS using both TCGA-COAD MSI samples and TCGA-COAD NAT samples. We choose 24 genes from the KEGG Wnt signalling pathway.

*SLC2A1 regulation in colon cancer cell lines* [62] found that the effectors of Wnt signalling $\beta$-catenin (CTNNB1) and TCF7L2 in collaboration with ZIC proteins directly regulate SLC2A1 gene expression. We choose gene expression of 14 genes from the KEGG Wnt signalling pathway together with ZIC5 and SLC2A1 genes and observe any difference in SLC2A1 regulation between ZIC5 WT and ZIC5 KO samples. GSE127960 data set is used to assess the IntOMICS performance only at the gene expression level.

*CNVs specific for MSS colon cancer* The TCGA colon cancer (TCGA-COAD) microsatellite stable (MSS) samples were used to evaluate IntOMICS ability to infer dependencies among different molecular features—gene expression and copy number variation. In this part, we utilise gene expression, DNA methylation, and CNV data of TCGA-COAD MSS stage II/III samples (n = 27). We choose a subset of 24 genes from the KEGG Colorectal cancer pathway concerning CNVs identified by [54] in MSS stage II/III primary tumours. [54] identified several MSS specific aberrations. We focus on amplification of KRAS, MYC, BIRC5, CCND1, RAC3 and deletion of SMAD4.

*DNA methylation specific for MSI colon cancer* The TCGA colon cancer (TCGA-COAD) microsatellite instable (MSI) samples have characteristic molecular biomarkers such as gene expression silencing through the MLH1 promoter hypermethylation [63, 64]. TCGA-COAD MSI samples were used to evaluate IntOMICS ability to infer dependencies among other molecular features—gene expression and DNA methylation. In this part, we utilise gene expression, DNA methylation, and CNV data of TCGA-COAD MSI samples (n = 69). We choose a subset of 24 genes from the KEGG Colorectal cancer pathway and MLH1. Because there are many methylation probes with significant regression coefficient and $R^2 > 0.3$, we perform differential methylation analysis using ChAMP R-package [65]. We select CpG island methylation probes from the promoter region with differential methylation between NAT and MSI samples (*p*-value $< 0.05$), and the absolute value of delta beta was greater than its 0.75 quantile.

*In Silico Dataset* We use in silico dataset DREAM4 with the gold standard network structure available to evaluate the IntOMICS performance at the gene expression level. For each network, 50 % of the known interactions were randomly selected as the prior knowledge.

*Comparison with Algorithms for Multi*-*Omics* *Regulatory Network Inference* Finally, we compare IntOMICS to two existing approaches focused on modelling interactions between multi-*omics* modalities with available implementation, namely RACER [18] and KiMONo [21]. Both methods can predict the interaction between CNV/METH/miRNA and GE modalities. miRNA-GE interactions are excluded from this comparison. RACER can also predict GE-GE interactions restricted to TFs and their targets. KiMONo is designed to evaluate only GE-GE interactions listed in the prior knowledge. For this comparison, we use TCGA-AML (acute myeloid leukemia) dataset from the RACER publication [18] to reproduce the most relevant results. In this part, we focus on 25 genes from the Notch signalling pathway, that is crucial in malignant transformation and therefore extensively studied [66]. Two out of these 25 genes are known TFs with targets from Notch signalling pathway.

### Real application of InfOMICS: ABCG2-related chemoresistance in MSS stage III colon cancer

Resistance to chemotherapy is a major clinical problem in colon cancer treatment. Mechanisms associated with chemoresistance and novel biomarkers can identify patients with benefit from irinotecan-based regimens that could substantially improve the selection of cancer therapy for the individual patient.

PETACC-3 clinical trial [53] randomised colon cancer patients to fluorouracil/leucovorin (5FU/FA) $+/-$ irinotecan treatment groups. The combination of ABCG2 and TOP1 gene expression significantly divided the stage III colon cancer patients enrolled in PETACC-3 into two groups regarding benefit from adjuvant treatment with FOLFIRI [67].

ABCG2 plays an essential role as an efflux transporter with various substrates, including chemotherapy drugs. Thus it is responsible for chemotherapy failure [68]. MYCN (by analogy, c-MYC) can contribute to irinotecan chemoresistance by regulating a specific set of ABC transporter genes, including ABCG2. Direct interaction was determined by chromatin immunoprecipitation (ChIP) assays and luciferase-reporter assays [69]. The authors have shown that ABCG2 gene expression is positively regulated by MYCN in neuroblastoma cell lines.

The gene expression of ABCG2 could be increased by activation of mitogen-activated protein kinase cascade via phosphorylation of extracellular regulated kinase ERK1/2 and c-jun NH-terminal kinase/stress-activated protein kinase (JNK/SAPK) [70].

We use IntOMICS to investigate mechanisms associated with chemoresistance using 176 MSS stage III colon cancer samples from the PETACC-3 (PETACC-3 MSS) clinical trial [53, 54]. We select 37 genes from the KEGG MAPK signalling pathway together with MYCN and ABCG2 genes. Copy number variation data are available for 23 out of 39 selected genes. Both PETACC-3 MSS samples treated by 5FU/FA (PETACC-3 MSS 5FU/FA) and PETACC-3 MSS samples treated by 5FU/FA + irinotecan (PETACC-3 MSS FOLFIRI) are dichotomized by 5-year relapse-free survival (RFS) into high/low-RFS groups (Table 3).

**Table 3:** Number of samples in PETACC-3 clinical trial according to the treatment and relapse-free survival

	5FU/FA	FOLFIRI
Low-RFS	31	34
High-RFS	53	58

*5FU/FA* fluorouracil/leucovorin, *FOLFIRI* fluorouracil/leucovorin with irinotecan, *RFS* relapse-free survival

## Results

**Fig. 2:**
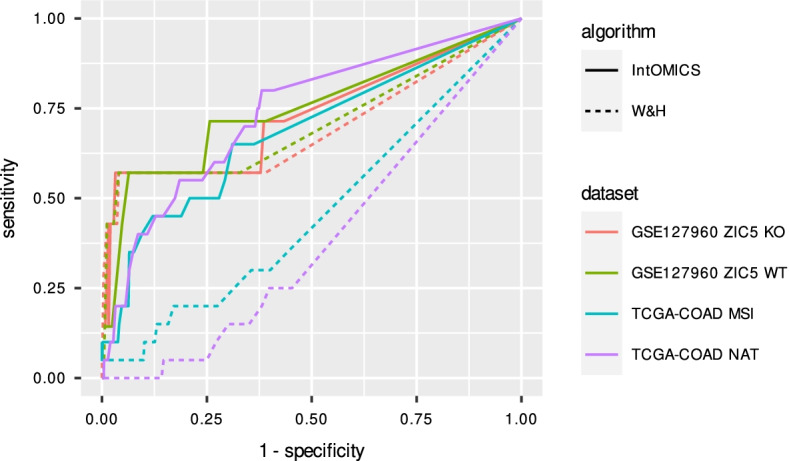
Performance comparison of IntOMICS and W &H algorithm [4] using real datasets. Receiver-operating characteristic curve (as a function of the edge weights) serves as the main performance index. Gold standard Wnt signalling pathway is used as the ground truth. NAT histologically normal tissue adjacent to the tumour; MSI microsatellite instability; WT wild-type; KO knockout

The performance of IntOMICS and W &H algorithm in TCGA-COAD and GSE127960 datasets is shown in Fig. 2. Corresponding AUC with 83% CIs, Cohen’s $\kappa$ coefficient, and running time can be found in Additional file 2: Table S1. In TCGA-COAD NAT and TCGA-COAD MSI datasets, the IntOMICS performance is significantly higher than the W &H. There is no statistically significant difference in the performance in GSE127960 ZI5 WT and GSE127960 ZI5 KO datasets.

Cohen’s $\kappa$ ranges between 0.12 to 0.38. In the TCGA-COAD datasets, Cohen’s $\kappa$ is very low, which reflects the statistically significant difference in the performance. In the GSE127960 ZIC5 WT dataset, Cohen’s $\kappa = 0.19$ is also low, even if the performance of both algorithms is similar. However, we can notice the lack of convergence in W &H MCMC simulation. As a result of the Markov chain poor convergence, we can detect apparent anomalies in the trace plots of $\beta$ values (Additional file 1: Fig. S3a, b) and inconsistency of posterior probabilities of the edges (Additional file 1: Fig. S4a, b). [4] decided to run a fixed number of MCMC simulations for performance evaluation. We followed the same procedure in the W &H application and found out that this setting is insufficient in some datasets. Consequently, using a measure of convergence, such as the $c_{\mathop {rms}}$ [38], is necessary for every MCMC simulation. IntOMICS trace plots of $\beta$ values and the edge posterior probabilities of two independent MCMC simulations can be found in Additional file 1: Figs. S5 and S6.

$\beta$-*catenin regulation in MSI colon cancer* The W &H algorithm identified the interaction from CTNNB1 to GSK3$\beta$ in both TCGA-COAD NAT and TCGA-COAD MSI datasets. Using the predefined threshold of edge weights, the interaction from GSK3$\beta$ to CTNNB1 was missing in the resulting regulatory network derived by IntOMICS in TCGA-COAD NAT samples. The interaction was present in 50% of CPDAGs. In TCGA-COAD MSI samples, IntOMICS did not identify the interaction between these genes at all, even if $B_{ij} = 1$ for i = GSK3$\beta$ and j = CTNNB1. This result suggests IntOMICS can discover relevant data-derived interactions despite distinct prior knowledge. We can conclude the edge weight needs to be considered carefully when interpreting the results and drawing conclusions.

In the TCGA-COAD NAT dataset, 17% and 13% of edges identified exclusively by the W &H and IntOMICS algorithm were found in other databases, respectively.

*SLC2A1 regulation in colon cancer cell lines* Based on the [62] experiment, we assume SLC2A1 is directly regulated by ZIC5, CTNNB1, and TCF7L2 in ZIC5 WT samples ($B_{ij} = 1$).

In ZIC5 WT samples, both W &H and IntOMICS revealed all three interactions. Moreover, IntOMICS revealed GSK3B as the direct regulator of SLC2A1 and TCF7L2 as the direct regulator of ZIC5. W &H revealed CTNNB1 as the direct regulator of ZIC5.

We expect a difference in SLC2A1 and ZIC5 regulation in ZIC5 KO samples. In ZIC5 KO samples, W &H revealed the same set of SLC2A1 regulators. The difference is missing interaction between CTNNB1 and ZIC5. IntOMICS identified a direct regulation from CTNNB1, TCF7L2, and CHD8 to SLC2A1. IntOMICS identified the same interaction from TCF7L2 to ZIC5 as in WT samples.

In the GSE127960 WT dataset, 7% and 18% of edges identified exclusively by the W &H and IntOMICS algorithm were found in other databases, respectively.

*CNVs specific for MSS colon cancer* IntOMICS identified edges from CNV to associated GE in five out of six genes of interest: KRAS, MYC, BIRC5, RAC3, and SMAD4. Even if the interaction from CCND1 CNV to CCND1 GE is not present in the resulting network structure, the edge weight is higher than the median of all edge weights in the resulting network.

Except five genes mentioned above, we should highlight other interesting interactions identified by IntOMICS: deletion of tumour suppressor SMAD2 directly connected with SMAD2 GE, amplification of proto-oncogene BRAF directly connected with BRAF GE, two DNA methylation probes located at the CpG island shore (2-kb-long region from CpG island) directly connected with FOS GE, and one DNA methylation probe directly connected with TGFBR2 GE. IntOMICS also identified interesting interplay between PIK3R5 CNV, one methylation probe located at CpG island of PIK3R5 and PIK3R5 GE. PIK3R5 was previously found to be mutated in colon cancer [71, 72].

The resulting regulatory network inferred by the IntOMICS algorithm using TCGA-COAD MSS stage II/III samples can be seen in Additional file 1: Fig. S7.

*DNA methylation specific for MSI colon cancer* IntOMICS identified six DNA methylation probes (all located at CpG island) as MLH1 direct regulators. There are no descendant nodes of the MLH1 gene. This is in concordance with the hypothesis that DNA methylation of the MLH1 promoter region influences its gene expression.

The resulting regulatory network inferred by the IntOMICS algorithm using TCGA-COAD MSI samples can be seen in Additional file 1: Fig. S8.

**Fig. 3:**
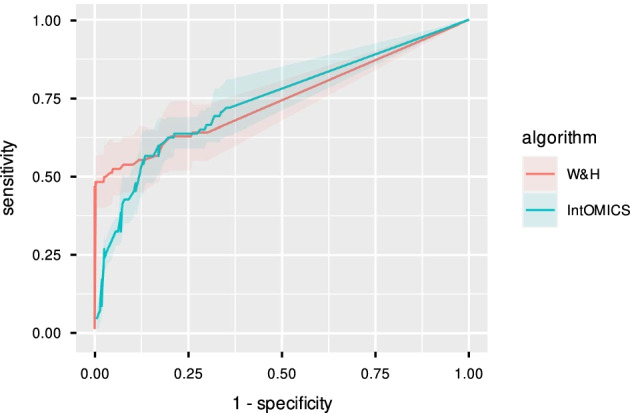
Performance comparison of IntOMICS and W &H algorithm [4] using in silico **gene expression dataset.** Receiver-operating characteristic curve (as a function of the edge weights) with 95% confidence interval serves as the main performance index

*In Silico Dataset* IntOMICS and W &H performance using DREAM4 in silico gene expression dataset in terms of ROC is shown in Fig. 3. The difference of IntOMICS AUC = 0.74, 83% CI = (0.71–0.78) and W &H AUC = 0.75, 83% CI = (0.71–0.79) is not statistically significant. We can see slightly different sensitivity in the region of high specificity. However, the sensitivity is balanced if specificity decreases to 90%. Cohen’s $\kappa$ ranges between 0.22 to 0.59 (see Additional file 2: Table S1).

Presented results indicate a difference in favour of IntOMICS, and we can conclude that the proposed framework can compete with the W &H algorithm using only gene expression data.

**Fig. 4:**
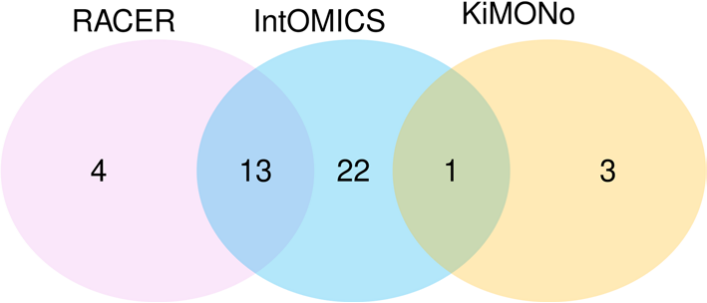
The intersection of interactions between features originating from distinct omics modalities identified by RACER, KiMONo, and IntOMICS

*Comparison with Algorithms for Multi*-*Omics* *Regulatory Network Inference* Both RACER and KiMONo tested (i) 50 interactions between CNV/METH and corresponding GE, and (ii) 11 interactions between TFs and their targets. On top of that, KiMONo allows CNV/METH to be a gene expression regulator of any other gene. RACER identified 17/50 interactions between CNV/METH and corresponding GE as significant. KiMONo identified 1/50 interactions between CNV/METH and corresponding GE as significant. On top of that, KiMONo identified another three interactions between CNV/METH and GE that were not tested by RACER or IntOMICS. Venn diagram of interactions between features originating from distinct *omics* modalities identified by RACER, KiMONo, and IntOMICS is shown in Fig. 4. RACER identified 3/11 interactions between TFs and their targets as significant (one of them was identified also by IntOMICS). KiMONo identified 1/11 interactions between TFs and their targets as significant (IntOMICS identified this interaction in the opposite direction).

KiMONo is also designed to test GE-GE interactions defined in the prior knowledge. We used 24 known GE-GE interactions from the KEGG Notch signalling pathway as the prior knowledge. KiMONo identified 1/24 GE-GE interactions from the prior knowledge as significant (from NOTCH3 GE to NCSTN GE). IntOMICS identified also 1/24 GE-GE interactions from the prior knowledge (from MAML2 GE to RBPJL GE).

These results suggest there is some overlap between these algorithms, especially between RACER and IntOMICS. However, all these methods have its disadvantages over the others. RACER does not test any GE-GE interactions except TFs and their targets. KiMONo does not test any GE-GE interactions except the prior knowledge. KiMONo also derived several CNV/METH-GE interactions that are not straightforward to interpret, such as interaction from NOTCH3 CNV to APH1B GE. IntOMICS requires considerable time complexity and is limited by the number of input features. Nevertheless, IntOMICS provides the best choice for detailed knowledge discovery from multi-*omics* data.

### Real application of InfOMICS: ABCG2-related chemoresistance in MSS stage III colon cancer

There are several interesting differences in ABCG2 regulation in the PETACC-3 MSS samples. Genes with ABCG2 direct interaction determined by IntOMICS are listed in Table 4. In all PETACC-3 MSS samples, IntOMICS identified direct interaction from ABCG2 CNV to ABCG2 GE.

**Table 4:** Genes with ABCG2 direct interaction and the confidence of the regulation (*w*) determined by IntOMICS

	5FU/FA		FOLFIRI	
	Low-RFS	High-RFS	Low-RFS	High-RFS
ELK1	High	Med	Low	Med
HRAS	Low	Med	Low	High
MAP3K1	High	Med	Low	NA
MAP2K1	Med	High	NA	Low
MAPK1/ERK2	Low	Low	Med	High
MAPK3/ERK1	High	Low	Low	NA
MRAS	Low	Low	High	Low
MYC	High	Med	Med	Med
MYCN	Low	Low	Low	High
RAF1	Med	NA	Low	High
RPS6KA3	High	NA	NA	Low
ABCG2 CNV	High	High	High	High

Genes with the highest predictive potential are highlighted in bold. low $w < 0.5$ quantile of all edge weights; med $0.5 \ge w < 0.75$ quantile of all edge weights; high empirical frequency $w \ge 0.75$ quantile of all edge weights; NA the edge was not identified

MAPK3/ERK1 and RPS6KA3 genes are identified as the direct regulators of the ABCG2 gene in the 5FU/FA low-RFS samples. In contrast, the confidence of ABCG2 regulation by these genes is very low in other groups. These results support the findings of [70] about the regulation of ABCG2 by MAPK3/ERK1, which can have therapeutic consequences.

MRAS is identified as the direct regulator of the ABCG2 gene only in the FOLFIRI low-RFS samples (the confidence of MRAS GE and ABCG2 GE direct regulation is very low in other groups).

MYCN is identified as the direct regulator of the ABCG2 gene in the FOLFIRI high-RFS samples (the confidence of MYCN GE and ABCG2 GE direct regulation is very low in other groups). ABCG2 contributes to chemoresistance through the efflux of anticancer drugs from cancer cells [73] and MYCN was shown to be positive regulator of ABCG2 [69]. We can hypothesise that the direct interaction between MYCN GE and ABCG2 GE in the group of FOLFIRI high-RFS samples does not necessarily lead to irinotecan resistance development. We do not observe statistically significant difference in MYCN GE or ABCG2 GE between groups (Kruskal-Wallis test; *p*-value $\ge$ 0.1). On the contrary, the Spearman’s corelation coefficient test between ABCG2 GE and MYCN GE is at the margin of statistical significance in the FOLFIRI high-RFS samples and the correlation coefficient is positive in comparison with other groups (see Table 5). The role of MYCN in ABCG2-related chemoresistance remains uncertain.

**Table 5:** Spearman’s correlation coefficient between ABCG2 GE and MYCN GE in MSS stage III colon cancer and corresponding *p*-value

Treatment and survival	$\rho$	*p*-value
5FU/FA low-RFS	− 0.17	0.35
5FU/FA high-RFS	− 0.11	0.41
FOLFIRI low-RFS	− 0.01	0.96
FOLFIRI high-RFS	0.23	0.09

$\rho$ Spearman’s correlation coefficient; *5FU/FA* fluorouracil/leucovorin, *FOLFIRI* fluorouracil/leucovorin with irinotecan, *RFS* relapse-free survival

In our study, MAPK3/ERK1, MRAS, MYCN, and RPS6KA3 have the highest predictive potential.

The direct interaction from MAPK1/ERK2 to ABCG2 is detected in FOLFIRI high-RFS samples. In the context of ABCG2 regulation, we can speculate about the functional redundancy of MAPK1/ERK2 and MAPK3/ERK1.

## Discussion

We present IntOMICS, a Bayesian framework for multi-*omics* data and prior knowledge integration to infer regulatory networks using a novel approach to biological knowledge discovery. Besides the integration of known interactions as prior knowledge, IntOMICS complements the prior knowledge using *empirical* biological matrix, which reflects hidden patterns in the available datasets. IntOMICS is designed to infer not only dependencies among gene expression but also between gene expression, DNA methylation and copy number variation. Pathogenic copy number variations and epigenetic changes (such as DNA methylation) can affect gene expression, contribute to increased DNA instability and play an essential role in the initiation and progression of complex diseases such as cancer. The great benefit of IntOMICS is the use of continuous data. Because frequently used data discretisation in multi-*omics* data analysis implies substantial information loss. The proposed framework minimises the weaknesses of MCMC-based algorithms utilising state-of-the-art approaches such as Markov blanket resampling or adaptive MCMC algorithm. IntOMICS can be extended with any additional modality if the proposed model assumptions are satisfied (variables come from the multivariate Gaussian distribution).

Although IntOMICS evaluation and application is demonstrated using *multi*-omics data of colon cancer samples, it is not limited to any particular phenotype.

We have to mention that IntOMICS is not designed to infer genome-wide regulatory networks because of time complexity. At present, IntOMICS is restricted to infer regulatory networks within pathways with up to tens of features (nodes).

The main limitations of IntOMICS are time complexity and limited flexibility of the linear model. Therefore, the maximal number of parents for a node is limited. The BGe score does not provide such modelling flexibility and enables the modelling of only linear relationships between features. However, relationships in biological systems are more variable and complex. For example, co-regulation of a given gene by two exclusive regulators cannot be captured with a linear model. Moreover, Bayesian networks are also restricted to acyclicity and no feedback loops, common biological features. The user must always consider these limitations during the interpretation of the IntOMICS results.

Our Bayesian network-based framework tuned for gene expression, copy number variation, and DNA methylation is designed to work on any modalities defined in a continuous domain. However, IntOMICS is designed to infer regulatory network, even if copy number variation or DNA methylation data (or both) are not available.

Regarding future work, our Bayesian network-based framework could be extended by additional omics data types, such as miRNAs. Our next objective is to upgrade the proposed workflow to infer regulatory networks with an extensive set of features.

At the gene expression level, the performance of IntOMICS is comparable with a published algorithm based on Bayesian networks using both real and in silico datasets. In the context of multi-*omics* data, IntOMICS performance is significantly better in comparison with a published algorithm based on Bayesian networks. The ability to capture relevant crosstalks between copy number variation and gene expression is verified using known associations between copy number variation and gene expression in microsatellite stable stage II/III colon cancer samples. IntOMICS identified five out of six known associations. Microsatellite instable samples were used to verify crosstalks between gene expression and methylation. IntOMICS identified six DNA methylation probes as MLH1 direct regulators together with associated CNV.

Additionally, IntOMICS performance was compared with two algorithms for multi-*omics* regulatory network inference that can also incorporate prior knowledge in the inference framework. There is overlap of interactions between features originating from distinct *omics* modalities. However, all these algorithms have their advantages over the others. IntOMICS should be used if we are interested in detailed knowledge discovery from multi-*omics* data. Besides inferring relevant crosstalks between multi-*omics* modalities, IntOMICS is designed to capture also interactions within gene expression.

Using our novel framework, several ABCG2 regulator genes are discovered as potential predictive markers in microsatellite stable stage III colon cancer samples. However, all regulatory relationships discovered by IntOMICS need to be verified using more refined approaches.

IntOMICS is a powerful resource for exploratory systems biology and can provide valuable insights into the complex mechanisms of biological processes that has a vital role in personalised medicine.

## Supplementary Information

**Additional file 1: Fig. S1.** Different IntOMICS hyperparameters setting in DREAM4 in silico dataset and the resulting ROC with 95% confidence intervals. **Fig. S2.** Different IntOMICS hyperparameters setting in DREAM4 in silico dataset and the resulting AUC with 83% confidence intervals. **Fig. S3.** Trace plot of β values using the W&H algorithm. **Fig. S4.** Consistency in the marginal posterior probabilities of the edges using the W&H algorithm. **Fig. S5.** Trace plot of β values using the IntOMICS algorithm. **Fig. S6.** Consistency in the marginal posterior probabilities of the edges using the IntOMICS algorithm. **Fig. S7.** CNVs specific for MSS colon cancer investigation and the resulting regulatory network inferred by IntOMICS algorithm using TCGA-COAD MSS stageII/III samples. **Fig. S8.** DNA methylation specific for MSI colon cancer investigation and the resulting regulatory network inferred by IntOMICS algorithm using TCGA-COAD MSI samples.

**Additional file 2. Table S1.** The performance of IntOMICS and W&H algorithm: corresponding AUC with 83% CIs, Cohen's Kappa coefficient, and running time.

## Data Availability

Source code and data used for producing the presented results are available at: https://gitlab.ics.muni.cz/bias/intomics. KEGG pathways used in the current study are publicly available and were downloaded from the KEGG pathway database (https://www.genome.jp/kegg/pathway.html). Known interactions between transcription factors and their targets were downloaded from the Harmonizome (collection of processed datasets; https://maayanlab.cloud/Harmonizome/dataset/ENCODE+Transcription+Factor+Targets).
