# Correction: Using empirical biological knowledge to infer regulatory networks from multi-*omics* data

**DOI:** 10.1186/s12859-022-04931-4

**Published:** 2022-09-15

**Authors:** Anna Pačínková, Vlad Popovici

**Affiliations:** 1 RECETOX, Faculty of Science, Masaryk University, Kotlarska 2, Brno, Czech Republic; 2 Faculty of Informatics, Masaryk University, Botanicka 68a, Brno, Czech Republic

## Correction to: BMC Bioinformatics (2022) 23:351 10.1186/s12859-022-04891-9

Following the publication of the original article [1], the authors identified that the funding note was incorrect. The correct funding note is given below.

Funding

This work was supported by Czech Science Foundation (GACR) through grant no. 19-08646S and the European Union’s Horizon 2020 research and innovation programme under Grant Agreement No. 825410.

The original article [1] has been corrected.
