# Supplementary material for: Using empirical biological knowledge to infer regulatory networks from multi-omics data
Source: BMC Bioinformatics. 2022 Aug 22;23:351. doi: 10.1186/s12859-022-04891-9 (PMC9396869; doi:10.1186/s12859-022-04891-9)
Supplement: Supplementary file 1 — Additional file 1: Fig. S1. Different IntOMICS hyperparameters setting in DREAM4 in silico dataset and the resulting ROC with 95% confidence intervals. Fig. S2. Different IntOMICS hyperparameters setting in DREAM4 in silico dataset and the resulting AUC with 83% confidence intervals. Fig. S3. Trace plot of β values using the W&H algorithm. Fig. S4. Consistency in the marginal posterior probabilities of the edges using the W&H algorithm. Fig. S5. Trace plot of β values using the IntOMICS algorithm. Fig. S6. Consistency in the marginal posterior probabilities of the edges using the IntOMICS algorithm. Fig. S7. CNVs specific for MSS colon cancer investigation and the resulting regulatory network inferred by IntOMICS algorithm using TCGA-COAD MSS stageII/III samples. Fig. S8. DNA methylation specific for MSI colon cancer investigation and the resulting regulatory network inferred by IntOMICS algorithm using TCGA-COAD MSI samples. [file 12859_2022_4891_MOESM1_ESM.pdf]

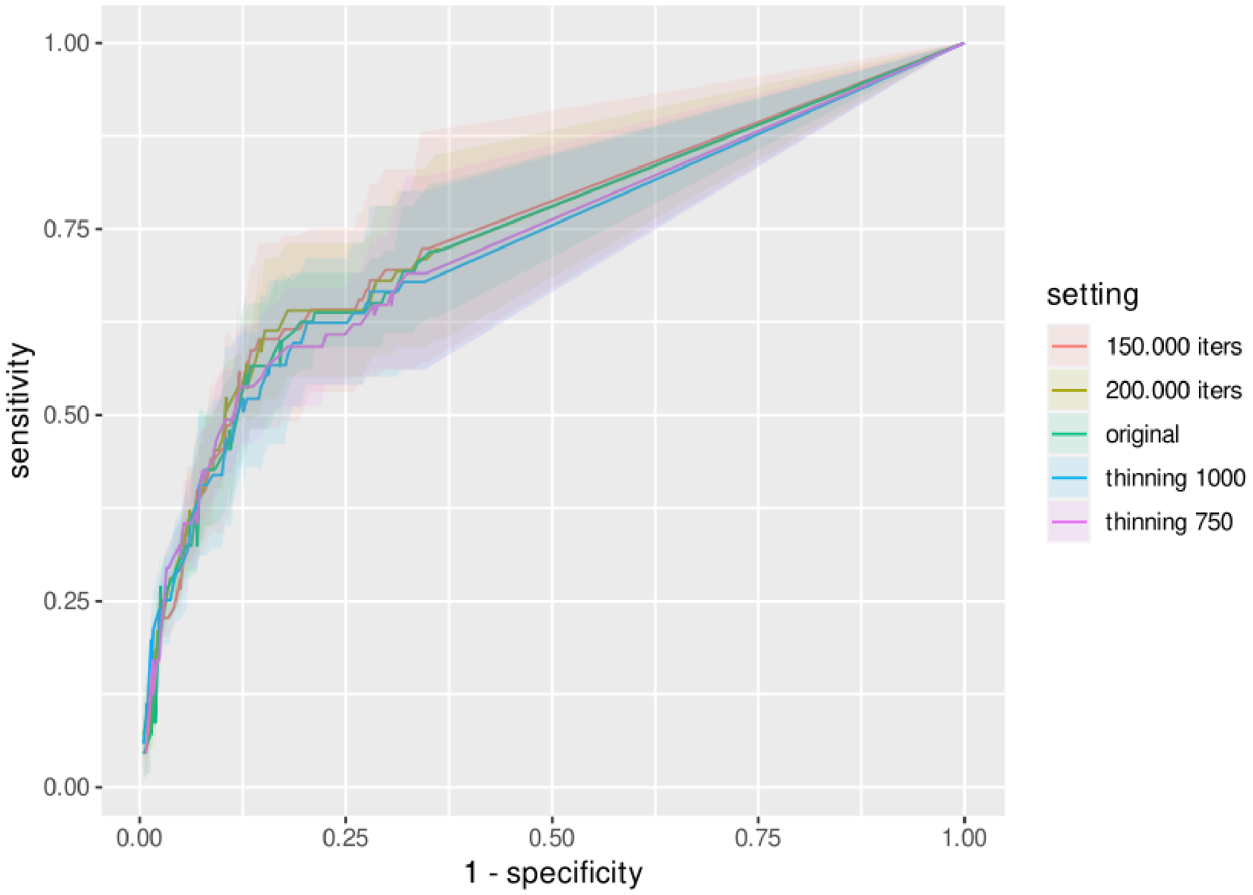

Supplementary Figure 1: Different IntOMICS hyperparameters setting in DREAM4 *in silico* dataset and the resulting ROC with 95% confidence intervals.

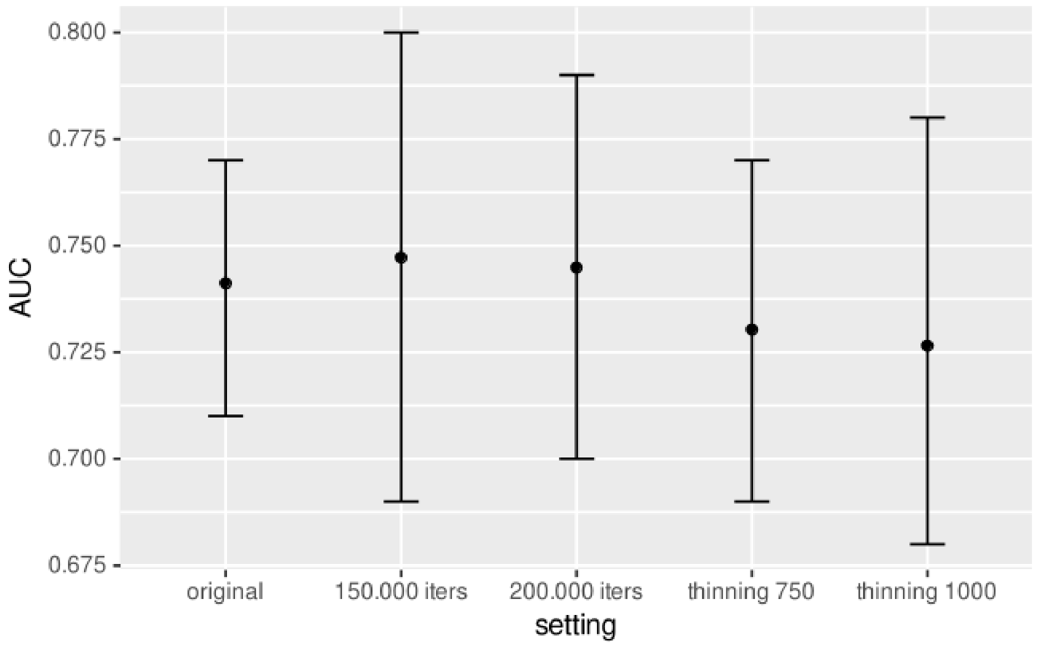

Supplementary Figure 2: Different IntOMICS hyperparameters setting in DREAM4 *in silico* dataset and the resulting AUC with 83% confidence intervals.

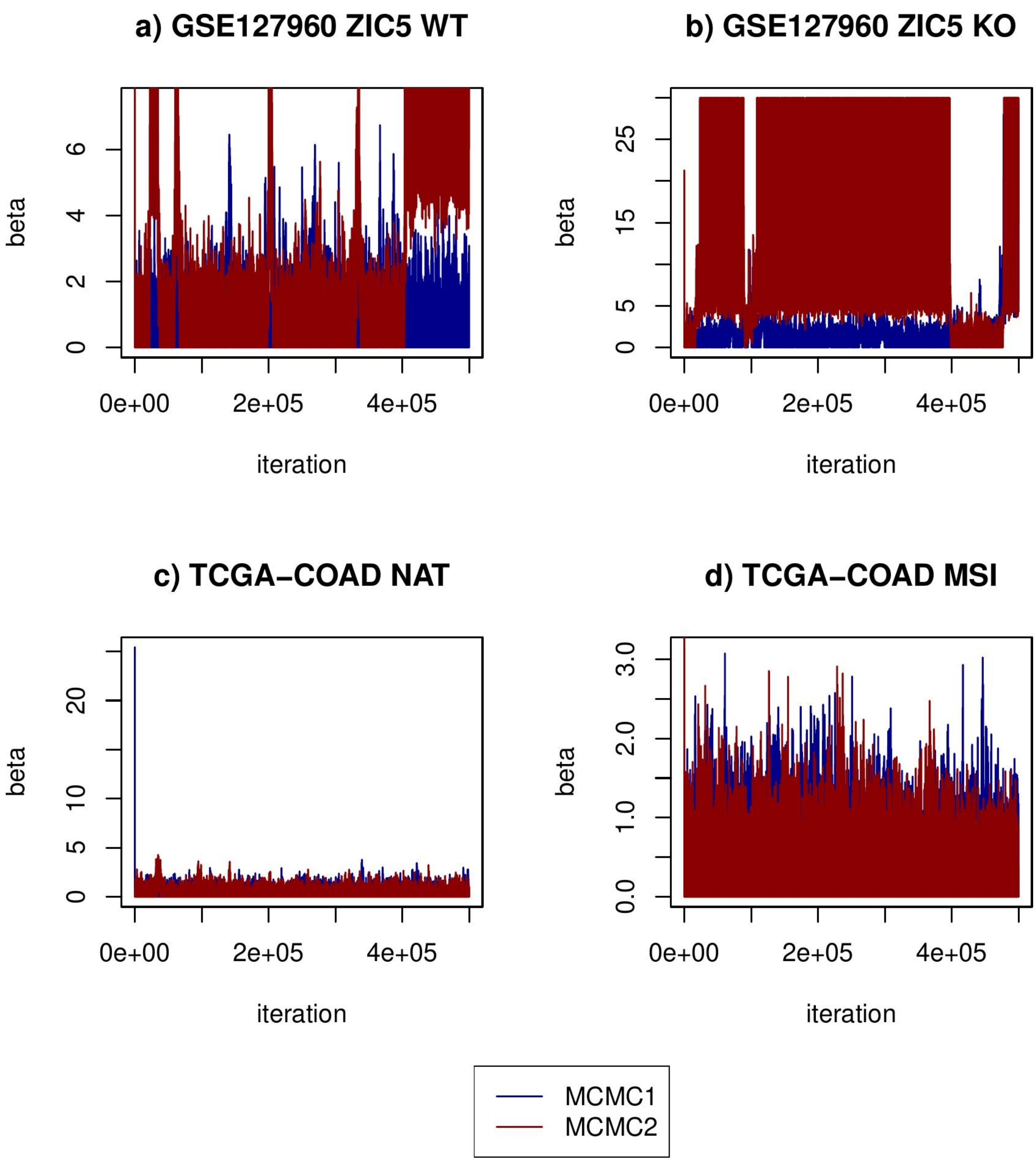

Supplementary Figure 3: Trace plot of β values using the W&H algorithm.

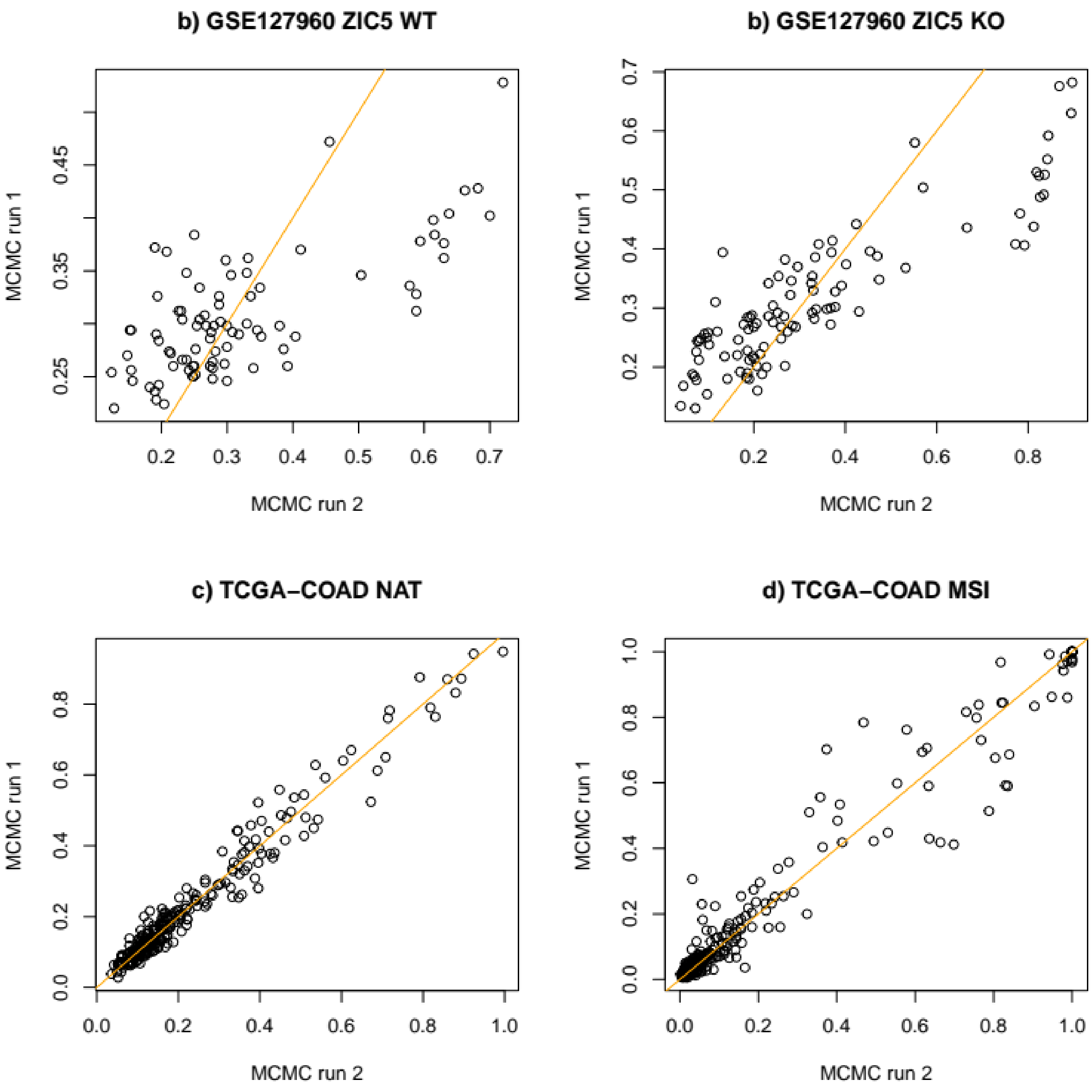

Supplementary Figure 4: Consistency in the marginal posterior probabilities of the edges using the W&H algorithm.

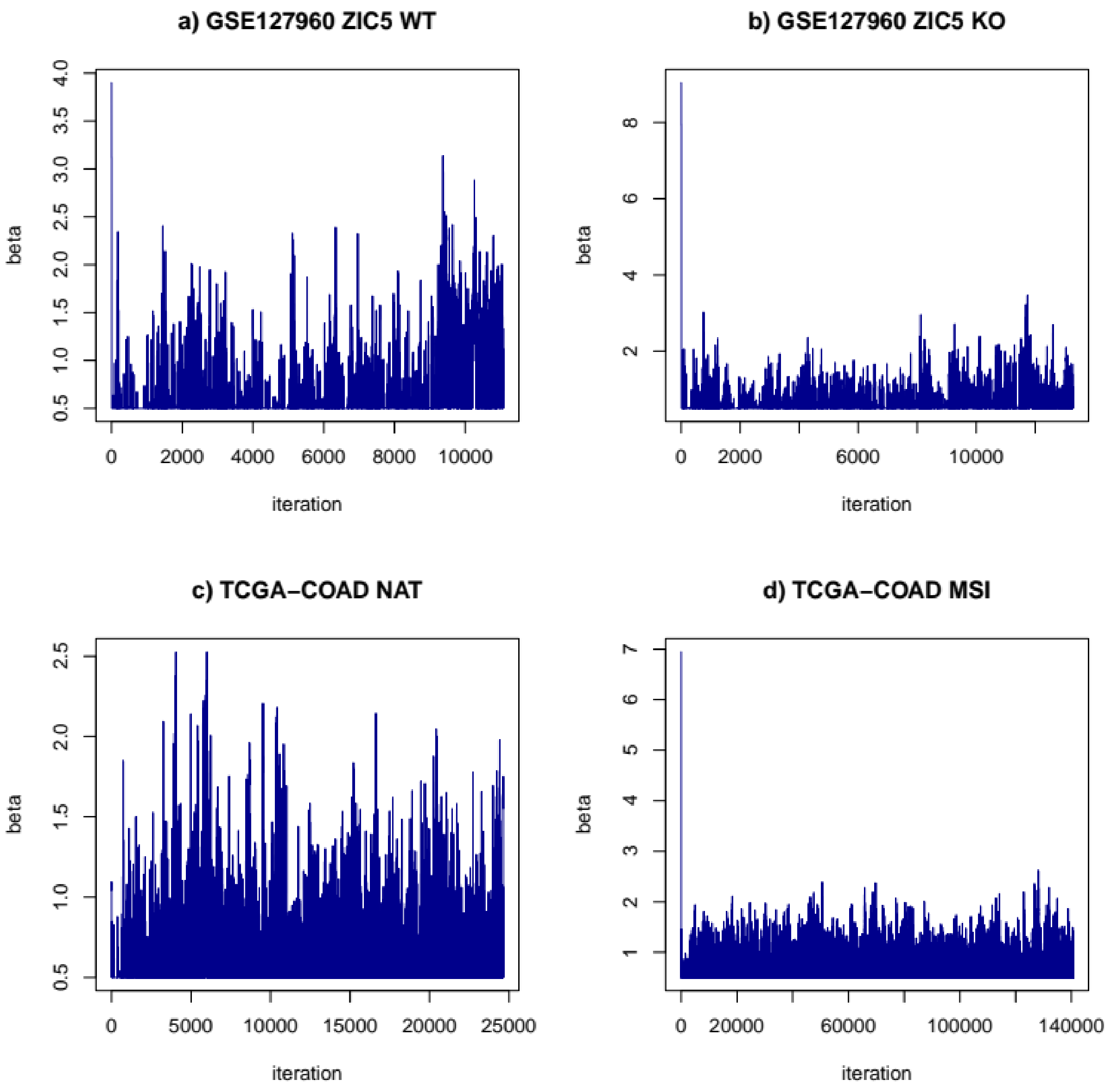

Supplementary Figure 5: Trace plot of β values using the IntOMICS algorithm.

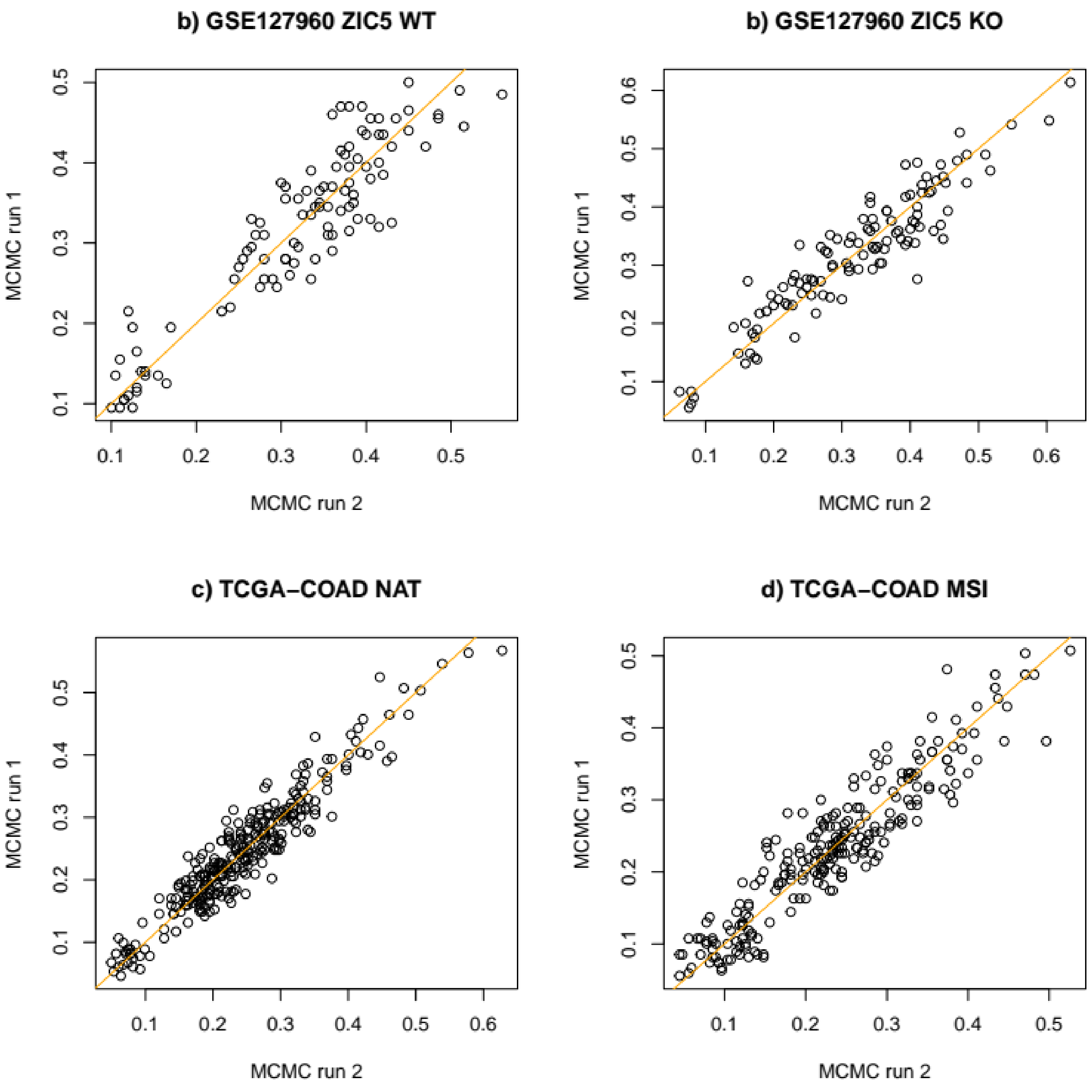

Supplementary Figure 6: Consistency in the marginal posterior probabilities of the edges using the IntOMICS algorithm.

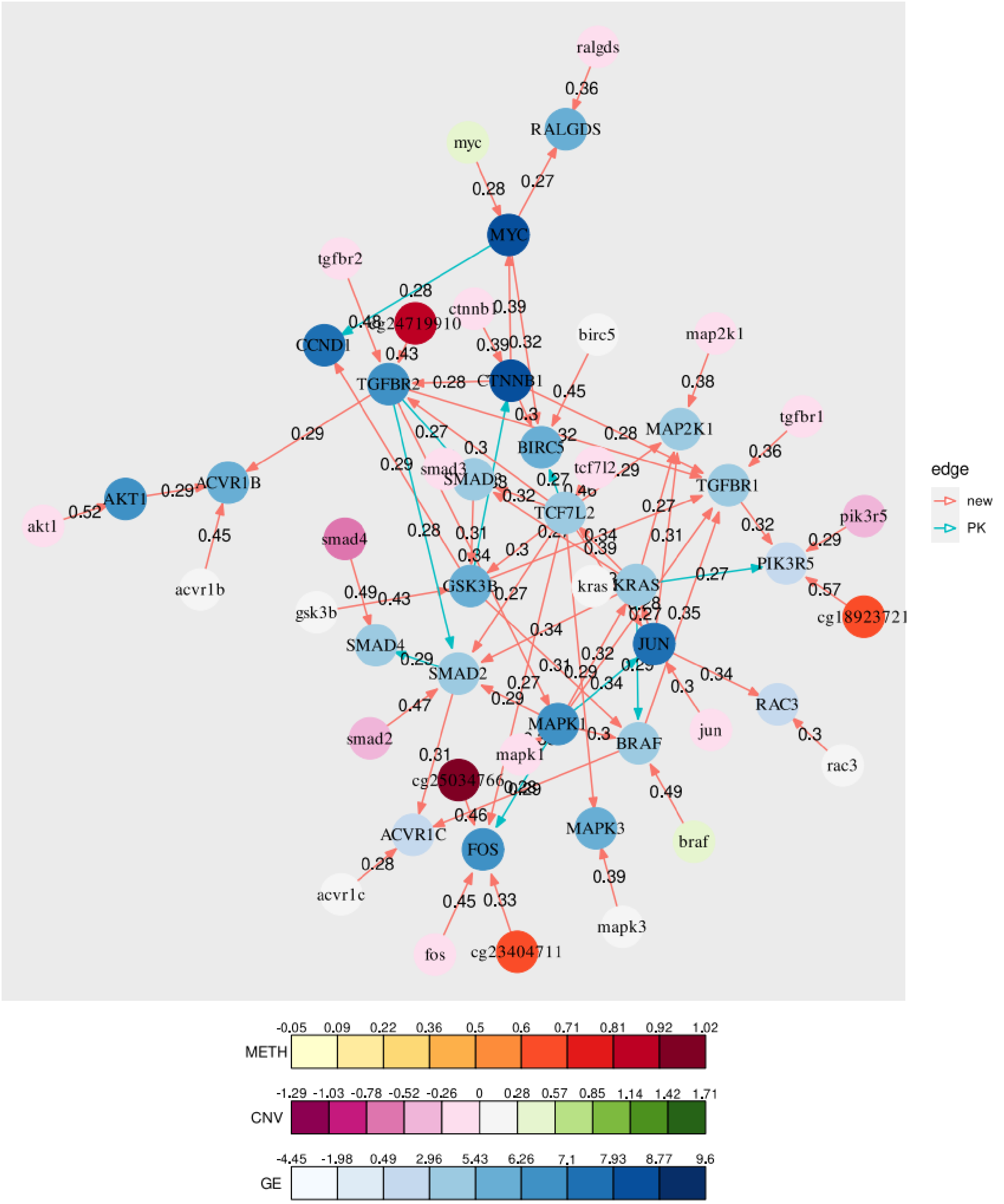

Supplementary Figure 7: CNVs specific for MSS colon cancer investigation and the resulting regulatory network inferred by IntOMICS algorithm using TCGA-COAD MSS stageII/III samples.

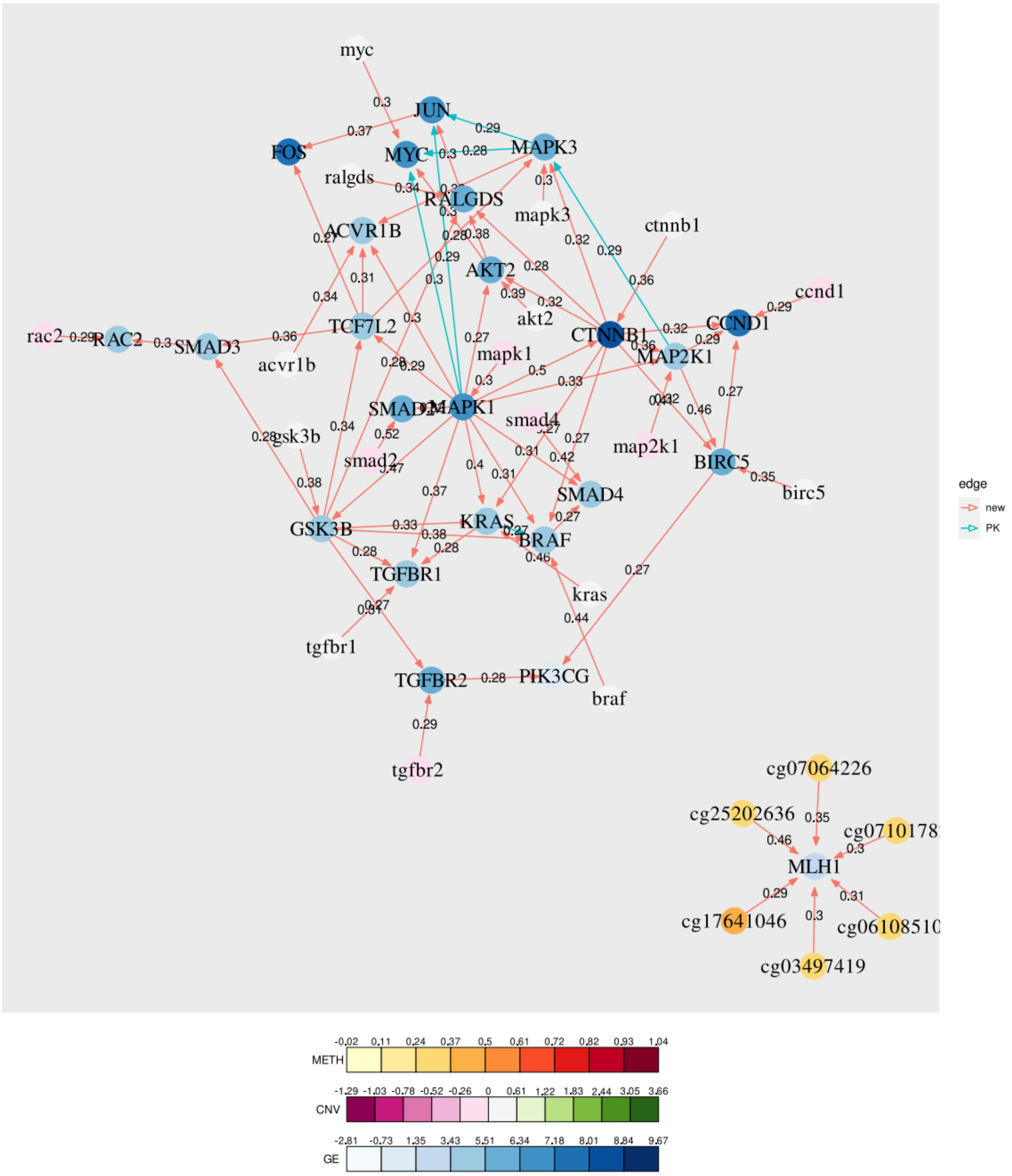

Supplementary Figure 8: DNA methylation specific for MSI colon cancer investigation and the resulting regulatory network inferred by IntOMICS algorithm using TCGA-COAD MSI samples.
