# Supplementary material for: Using empirical biological knowledge to infer regulatory networks from multi-omics data
Source: BMC Bioinformatics. 2022 Aug 22;23:351. doi: 10.1186/s12859-022-04891-9 (PMC9396869; doi:10.1186/s12859-022-04891-9)
Supplement: Supplementary file 2 — Additional file 2. Table S1. The performance of IntOMICS and W&H algorithm: corresponding AUC with 83% CIs, Cohen's Kappa coefficient, and running time. [file 12859_2022_4891_MOESM2_ESM.pdf]

The performance of IntOMICS and W&H algorithm: corresponding AUC with 83% CIs, Cohen's Kappa coefficient, and running time.

		TCGA-COAD					
		NAT	AUC 83% CI	Cohen's Kappa	MSI	AUC 83% CI	Cohen's Kappa
IntOMICS	# features	64	0.74	0.12	51	0.68	0.21
	time	57.12 h	(0.65;0.83)		30.24 h	(0.59;0.77)	
W&H	# features	24	0.38		24	0.46	
	time	28.08 h	(0.30;0.46)		15.11 h	(0.37;0.55)	

		GSE127960					
		WT	AUC 83% CI	Cohen's Kappa	KO	AUC 83% CI	Cohen's Kappa
IntOMICS	# features	16	0.75	0.19	16	0.73	0.38
	time	3.22 h	(0.6;0.9)		3.57 h	(0.58;0.88)	
W&H	# features	16	0.71		16	0.69	
	time	6.66 h	(0.56;0.86)		6.49 h	(0.54;0.84)	

		DREAM4									
		1	Cohen's Kappa	2	Cohen's Kappa	3	Cohen's Kappa	4	Cohen's Kappa	5	Cohen's Kappa
IntOMICS	# features	10	0.41	10	0.32	10	0.59	10	0.59	10	0.22
	time	1.29 h		1.78 h		1.32 h		1.32 h		1.56 h	
W&H	# features	10		10		10		10		10	
	time	2.34 h		2.16 h		2.29 h		2.27 h		1.99 h	
